# Caspase‐3‐mediated GSDME induced Pyroptosis in breast cancer cells through the ROS/JNK signalling pathway

**DOI:** 10.1111/jcmm.16574

**Published:** 2021-08-08

**Authors:** Ziwen Zhang, Han Zhang, Dongbo Li, Xiaoping Zhou, Qi Qin, Qingyuan Zhang

**Affiliations:** ^1^ Cancer Hospital of the University of Chinese Academy of Sciences (Zhejiang Cancer Hospital) Hangzhou China; ^2^ Institute of Cancer and Basic Medicine(IBMC) Chinese Academy of Sciences Hangzhou China; ^3^ Department of Medical Oncology Harbin Medical University Cancer Hospital Harbin Medical University Harbin China

**Keywords:** breast cancer, doxorubicin, GSDME, pyroptosis, ROS/JNK pathway

## Abstract

Pyroptosis is a new form of programmed cell death generated by some inflammasomes, piloting the cleavage of gasdermin (GSDM) and stimulation of dormant cytokines like IL‐18 and IL‐1β; these reactions are narrowly linked to certain diseases like diabetic nephropathy and atherosclerosis. Doxorubicin, a typical anthracycline, and famous anticancer drug has emerged as a prominent medication in several cancer chemotherapies, although its application is accompanied with expending of dose‐dependent, increasing, irreversible and continuing cardiotoxic side effects. However, the exact path that links the induced pyroptosis to the mechanism by which Doxorubicin (DOX) acts against breast cancer cells is still puzzling. The present study seeks to elucidate the potential link between DOX‐induced cell death and pyroptosis in two human breast cancer cell lines (MDA‐MB‐231 and T47D). We proved that treatment with DOX reduced the cell viability in a dose‐dependent way and induced pyroptosis morphology in MDA‐MB‐231 and T47D cells. Also, protein expression analyses revealed GSDME as a key regulator in DOX‐induced pyroptosis and highlighted the related role of Caspase‐3 activation. Furthermore, DOX treatments induced intracellular accumulation of ROS, stimulated the phosphorylation of JNK, and Caspase‐3 activation, subsequently. In conclusion, the study suggests that GSDME triggered DOX‐induced pyroptosis in the caspase‐3 dependent reactions through the ROS/JNK signalling pathway. Additionally, it showed that the DOX‐induced cardiotoxicity and pyroptosis in breast cancer cells can be minimized by reducing the protein level of GSDME; thus, these outcomes provide a new research target and implications for the anticancer investigations and therapeutic applications.

## 1 INTRODUCTION

Breast cancer is the most shared invasive cancer in women; this dangerous disease affects around 1 in 7 women in the world. Even though the incidence of breast cancer in East Asia is lower than in other regions of the world, the total number of patients is still very large due to its huge population (World Cancer Report 2003, International Agency for Research on Cancer (IARC). Because of the widespread physical examination and the specificity of its clinical symptom, breast cancer is often diagnosed at its early stage. As the cancer is promptly detected, patients can undergo surgery as a direct treatment with medications in addition.^1^, ^2^ Chemotherapy, one of the most common medications, is predominantly recommended for breast cancer patients in stages 2‐4. Most chemotherapy drugs kill fast‐growing or fast‐replicating breast cancer cells by causing DNA damage or other degradative mechanisms in cancer cells.^3^ However, in certain cases, the chemotherapy drugs may also damage normal breast cells and lead to serious side effects.^4^ Therefore, the precise mechanism by chemotherapy drug operates in cells demands deeper researches.

Pyroptosis is a highly inflammatory form of programmed cell death (PCD) that results from the activation of three different pathways. Firstly, the canonical inflammasome pathway, in which the activated Caspase‐1 cleaves the cytosolic gasdermin D (GSDMD) to forms transmembrane pores through the GSDMD N‐terminal (GSDMD‐N) and induces cell pyroptosis. The second mechanism involves the non‐canonical inflammasome pathway, induced by the direct binding of lipopolysaccharide (LPS) onto Caspase‐4/5 in humans. Finally, the third and the less‐studied pathway is the caspase‐3 dependent pyroptotic pathway, in which the Caspase‐3 may cleave the gasdermin E (GSDME‐N) to form transmembrane pores, then lead cells into pyroptosis process.^5^, ^6^

Doxorubicin (DOX) (commercial name: Adriamycin) is a common chemotherapy medication recommended for multiple cancer therapies, including breast cancer. DOX molecules interact with DNA by insertion and inhibition of macromolecular biosynthesis, and then stop the process of replication. It is applied with other drugs to form various chemotherapy units, such as AC (Adriamycin cyclophosphamide or Doxorubicin), TAC (Taxotere, Adriamycin, cyclophosphamide) and FAC (5‐fluorouracil, Adriamycin, cyclophosphamide).^7^ Recent studies on cisplatin‐DDP and doxorubicin‐induced pyroptosis revealed that caspase‐3‐mediated GSDME activation played a crucial role in mouse macrophages; moreover, a similar study demonstrated that DOX‐induced pyroptosis was generated through the ROS‐JNK‐caspase 3‐GSDME signalling in human tubular epithelial cells, suggesting GSDME as a potential target for chemotherapeutic drugs researches.^8^, ^9^ Meanwhile, different research showed that GSDMD exhaustion promptly stimulated the split of caspase‐3 and poly ADP ribose polymerase (PARP), and enhanced cancer cell death through mitochondrial pyroptosis pathways.^10^

Here, screening comparative analysis within the relative expression of GSDME in six different cell lines (BT549, T47D, MDA‐MB‐231, MDA‐MB‐468, MCF7 and MDA‐MB‐453) were conducted through qRT‐PCR essays, among which two of the most expressive cell lines were selected (MDA‐MB‐231 and T47D). Besides, we evaluated the induced cell death of six clinical chemotherapy drugs (PTX(Paclitaxel), DDP(Cisplatin), DOX(Doxorubicin), CTX(Cyclophosphamide) and 5‐FU(5‐fluorouracil), and the Doxorubicin treatment generated the severest pyroptosis reactions in both MDA‐MB‐231 and T47D cell lines. Furthermore, we investigated the involved pathway of DOX‐induced pyroptosis through the Western blotting assessments, and we found that, instead of GSDMD linked to most studied pyroptosis cases, the GSDME appeared to be the key regulator of the DOX‐induced cell death, surprisingly, while the protein expression of Caspase‐8 and Caspase‐3 were the most affected. These results demonstrated that DOX‐induced cardiotoxicity is related to the Caspase‐3‐dependent pyroptosis pathway. Our study provides new experimental evidence to the mechanism by which Doxorubicin mediates cell death and describes the DOX‐induced pyroptosis for the first time in breast cancer cells. From the perspective of ameliorating human cancer therapy, it is convenient to continue in‐depth study to find the mechanism and the relationship between pyroptosis and cell immune systems, and provide a new scheme for immunotherapy.

## 2 MATERIALS AND METHODS

### 2.1 Cell cultures

MDA‐MB‐231 and T47D cells gotten from Shanghai Institute of Cell Biology, Chinese Academy of Sciences were cultured in DMEM medium (Gibco) supplemented with 10% FBS (Gibco) and 10 U/mL Penicillin‐Streptomycin (Gibco), then, incubated at 5% CO_2_ at 37°C. Cells were passaged when reached 80% confluency. Once confluency reached 70%, then the cells were incubated in the presence or absence of PTX (Paclitaxel, 0.1 µmol/L, Shanghai Yuanye), DDP (Cisplatin, 50 µmol/L, Shanghai Yuanye, China), DOX (Doxorubicin, 1.1 µmol/L, Shanghai Yuanye), CTX (Cyclophosphamide, 200 µM, Shanghai Yuanye, China) and 5‐FU (5‐fluorouracil, 114 µM, Beijing Solarbio, China) and the Reactive oxygen species inhibitor NAC (N‐acetyl‐L‐cysteine, 5 mmol/L, Beijing Solarbio, China). All drugs were dissolved in DMSO (D5879, Sigma).

### 2.2 Real‐time quantitative PCR

Total RNA was extracted from BT549, T47D, MDA‐MB‐231, MDA‐MB‐468, MCF7, MDA‐MB‐453 using Trizol reagent (Thermo Fisher Scientific), and 1 μg of RNA was reverse transcribed to cDNA using reverse transcriptase (Vazyme). Real‐time quantitative PCR (RT‐PCR) was performed using the SYBR Green I real‐time detection kit (Cwbio, Beijing, China) on a CFX96 Detection System (Bio‐Rad). The relative gene expression was normalized to BT549. Primer sequences: GAPDH‐ Forward: TGTGGGCATCAATGGATTTGG; GAPDH‐ Reverse: ACACCATGTATTCCGGGTCAAT; GSDME‐Forward: TGCCTACGGTGTCATTGAGTT; GSDME‐Reverse: TCTGGCATGTCTATGAATGCAAA.

### 2.3 CRISPR‐Cas9 knockout and siRNA knockdown

We monitored the GSDME knockout cell lines by the CRISPR/Cas9 technology In brief, two single gRNAs (guide RNAs), sg1 5’‐CACCGAGCTGCCACCACCATTGCCT‐3’ and sg2 5’‐AAACAGGCAATGGTGGTGGCAGCTC‐3’ for targeting GSDME were cloned into the vector px458 with similar methods provided by previous work,^5^ and then, the constructed vector was named px458‐GSDME. MDA‐MB‐231 and T47D cells were seeded with a density of 1 × 10^5^ cells/well in 6‐well plates, and then transfected with 2 µg of px458‐GSDME plasmid by Lipofectamine 3000 (Invitrogen). After 48 hours, the stable GFP positive cells were sorted by BD FACSAria II cell sorter (BD Biosciences), and the single clones were sorted into a 96‐well plate for 2‐3 weeks upon the cell growth rate. Finally, the WB was applied to determine the protein expression of GSDME in each clone. For siRNA knockdown, siRNA Caspase‐8 (Cat#105023, Invitrogen) or control siRNA (Cat#4404020, Invitrogen) was transfected into the MDA‐MB‐231 and T47D cells with 70% cell confluence by using Lipofectamine RNAiMAX reagent (Invitrogen) according to the manufacturer's instructions. After 72 hours, transfected MDA‐MB‐231 and T47D cells were treated with drugs and subjected to subsequent analyses.

### 2.4 Cell viability assays

MDA‐MB‐231 and T47D cells were seeded into 96‐well culture plates with 3000 cells per well and treated with different doses and different drugs for 8 hours. Cell viability was detected with a microplate reader (Mannedorf, Switzerland) by using the Cell Counting Kit‐8 assay (Dojindo) according to the manufacturer's manual. Each experiment was repeated three times.

### 2.5 Pyroptosis evaluation by microscopy imaging

To examine the morphology of apoptotic and pyroptotic cells, cells were first seeded in 6‐well culture plates at about 40% cell confluency and then treated with different drugs. Static bright field images were captured using a Leica microscope. All image data representatives of at least three were randomly fields.

### 2.6 Protein extraction and Western blot

Cells were lysed in RIPA Lysis and Extraction Buffer (Cat#89900, Thermo, USA). Protein concentrations of the cell lysates were determined with Bradford Assay (Bio‐Rad). Protein lysates were then subjected to 4%‐20% Tris‐Glycine SDS PAGE and then transferred onto PVDF membranes. The membranes were blocked in 5% milk‐Tris‐buffered saline with 0.1% Tween (TBST) at 25°C for 1h and then incubated with primary antibodies at 4°C overnight. On the next day, the membranes were washed by TBST thrice before incubation with horseradish peroxidase‐conjugated secondary antibodies (Cell Signalling) at 25°C for 1 hour. The protein expression band was visualized by ECL chemiluminescence (Promega). Primary antibodies against GSDMD, GSDME and Caspase‐1/‐3/‐9 were purchased from Abcam (Cambridge, USA), antibodies against Caspase‐7/‐8 and β‐actin were purchased from Cell Signalling Technology, and antibodies against JNK and p‐JNK were purchased from Santa Cruz Biotechnology.

### 2.7 Measurement of ROS

The ROS levels were measured by a ROS Assay with DCFH‐DA (Beijing Solarbio). After treatment with drugs for 8 hours, cells were washed with PBS and stained with10 µM DCFH‐DA for 30 minutes at 37°C under dark conditions. The level of ROS was determined by FACSC calibur flow cytometer (BD Biosciences, USA).

### 2.8 Statistical analysis

Each experiment was repeated three times. Data are presented as the means ± SD. Student's *t* test or one‐way ANOVA was performed to compare the differences among the groups. Statistical analyses were performed with SPSS 18.0 software (SPSS Inc). *P* < .05 was considered statistically significant.

## 3 RESULTS

### 3.1 Doxorubicin induces cell death through pyroptosis in breast cancer cells

**FIGURE 1:**
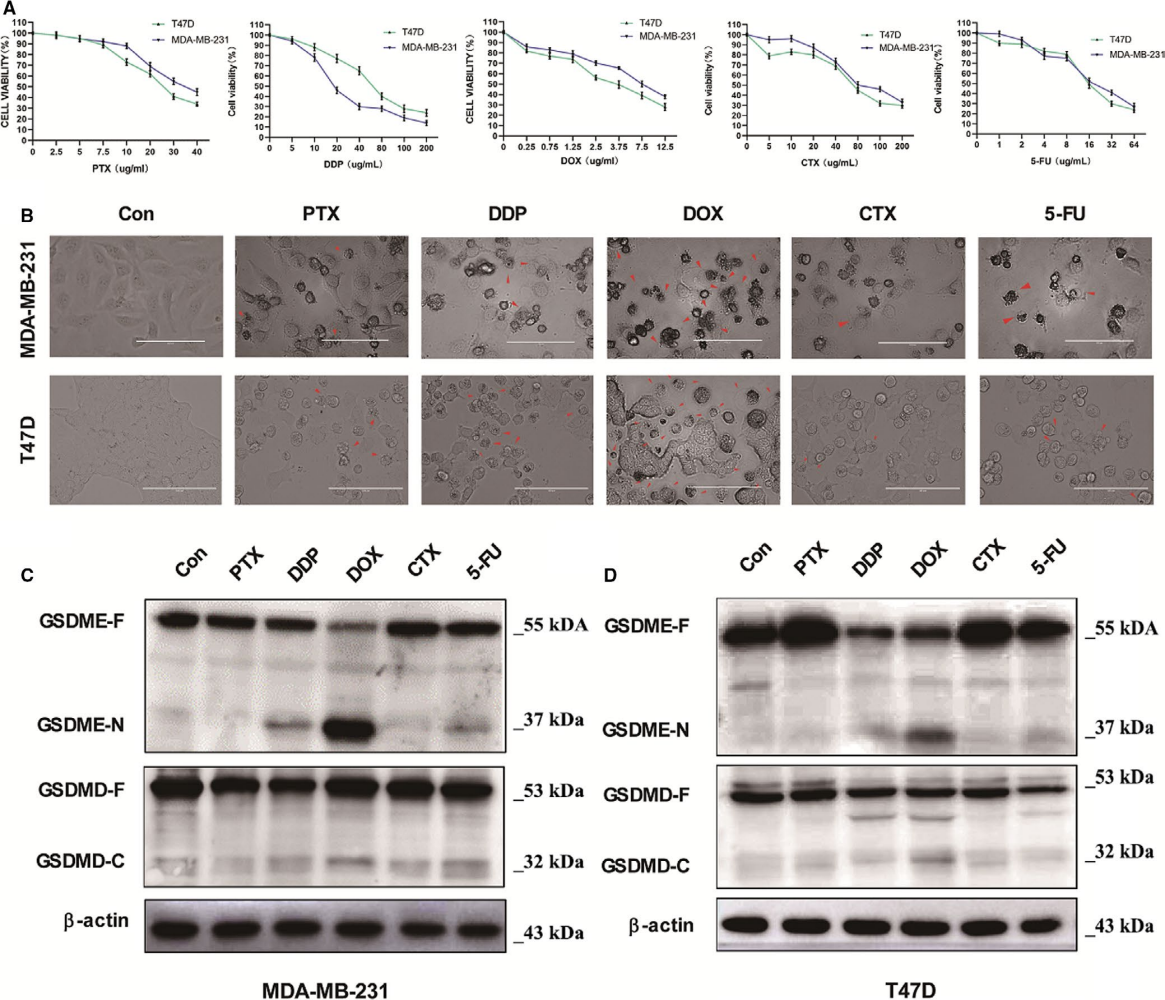
Chemotherapy drugs inhibit cell growth and induce cell pyroptosis in breast cancer cells. MDA‐MB‐231 and T47D cells were treated with different doses chemotherapy drugs for 12 h. (A) Cell viability of MDA‐MB‐231 and T47D was determined using the CCK8 assay. (B) Representative microscopic images of MDA‐MB‐231 and T47D after treated with PTX (0.1 µmol/L), DDP (50 µmol/L), DOX(1.1 µmol/L), CTX (200 µmol/L) and 5‐FU (114 µmol/L). Red arrowheads point to the characteristic balloon in the cell membrane. Scale bar, 25 µm. (C) and (D) Western blot images for the expression of GSDME and GSDMD with β‐actin as loading control for MDA‐MB‐231 and T47D cells

To evaluate the Doxorubicin drug effects and their induced pyroptosis on breast cancer cells, we compared the reactions of five common clinical chemotherapy drugs including PTX(Paclitaxel), DDP(Cisplatin), DOX(Doxorubicin), CTX(Cyclophosphamide) and 5‐FU(5‐fluorouracil) in two human breast carcinoma cell lines, MDA‐MB‐231 and T47D, and the CCK‐8 assays were performed to detect the cell viability (Figure 1). As predicted, the result showed that all chemotherapy drugs induced cell death in both MDA‐MB‐231 and T47D breast cancer cell lines. Curiously, the higher was the drug concentration, the smaller was the cell viability rate; this justified the hypothesis that chemotherapy drugs induce cell death in breast carcinoma cell lines in a dose‐dependent way (Figure 1A). In addition, cells were observed through a Leica microscope to judge the involved cell death reaction. Morphologically, all treated cells were releasing large bubbles from the plasma membrane, and knowing the fact that pyroptosis was defined in a previous study as the expansion of LDH release flanked by distinctive bubbles that emerged from the cell membrane, we concluded that drugs treatment had induced typical pyroptotic morphology (Figure 1B).^11^, ^12^ Meanwhile, DOX treatment showed the severest drug‐induced pyroptosis in both MDA‐MB‐231 and T47D breast cancer cell lines, followed by the DDP treatments.

### 3.2 GSDME cleavage is sufficient to mediate DOX‐induced pyroptosis

To verify which Gasdermin protein could be involved in drug pyroptosis‐induced in breast cancer cells, we assessed the protein expression of GSDME and GSDMD by Western blotting. We found that the protein expression of cleaved GSDME (GSDME‐N) was higher in both MDA‐MB‐231 and T47D breast cancer cell lines under DOX treatment, which is approximately consistent with the morphological results (Figure 1C). In contrast, the protein expressions of all cleaved GSDMD (GSDMD‐C) were minimized in all treated cells, with a slight augmentation in DOX treatment and stable expression under DDP (Figure 1C). Therefore, this couple of results ascertained that GSDME was the key instigator of DOX‐induced pyroptosis. In summary, we found that all chemotherapy drugs may lead to pyroptosis and GSDME cleavage in breast cancer cells. Also, DOX had the strongest ability to induce pyroptosis and the utmost GSDME cleavage among all drugs. Thus, the GSDME should be considered as the major regulator of pyroptosis‐induced cell death.^13^

### 3.3 Doxorubicin induces pyroptosis in the Caspase‐dependent pyroptosis pathway

**FIGURE 2:**
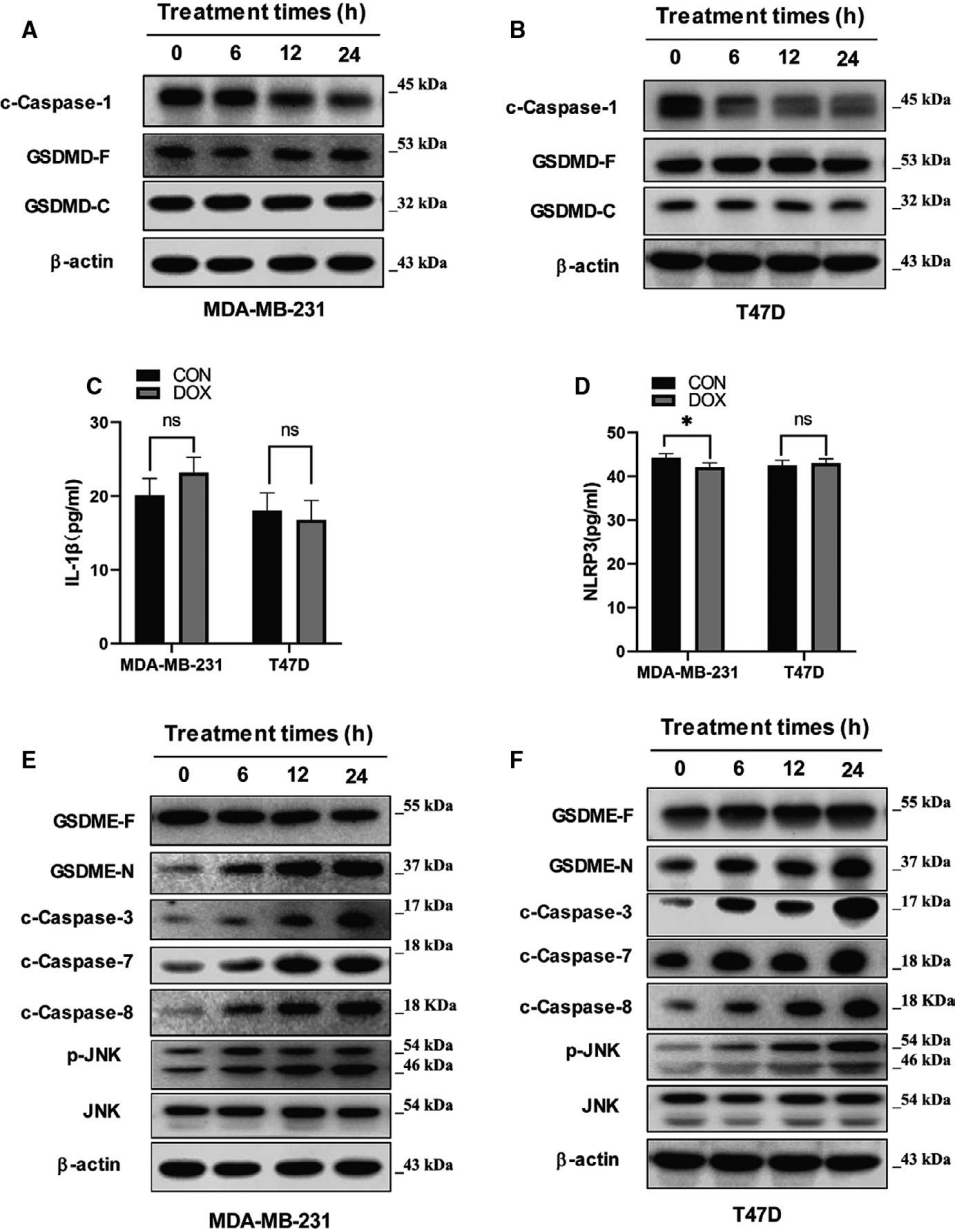
GSDME, rather than GSDMD, is cleaved during doxorubicin‐induced pyroptosis in breast cancer cells. (A) and (B) Western blot images for the expression of c‐Caspase 1 and GSDMD with β‐actin as loading control for MDA‐MB‐231 and T47D cells. (C) Protein level of IL‐1β in DOX treated cells. (D) Protein level of NLRP3 in DOX treated cells. (E) and (F) Western blot images for the expression of c‐Caspase 3 related protein and GSDME with β‐actin as loading control for MDA‐MB‐231 and T47D cells, respectively

Previous studies have exposed the split of Gasdermin family proteins as a pivotal reaction in cell pyroptosis, and that DOX treatment‐induced nephrotoxicity in cultured human renal tubular epithelial cells through Caspase 3/GSDME‐dependent pyroptosis.^11^ However, it is believed that pyroptosis reactions were stimulated and mediated by GSDMD/caspase‐1 which released pro‐inflammatory actors like, cytokines, NLRP3 and interleukin‐1β (IL‐1β and IL‐18).^14^, ^15^ Here, we sought to verify the specificity of the Caspase protein involved in DOX‐induced pyroptosis on breast cancer cells by evaluating the protein expression of NLRP3 and interleukin‐1β in both MDA‐MB‐231 and T47D cell lines. The Western blot results showed that the protein expression of c‐Caspase‐1 in cells under DOX treatment was lightly reduced within 24h, while that of both the GSDMD‐F and GSDMD‐C barely changed (Figure 2A,B). This result attested that DOX‐induced pyroptosis is not mediated by the GSDMD/Caspase‐1‐dependent canonical inflammasome pathway. Moreover, the expression pattern of IL‐1β and NLRP3 showed no significant changes between control and treated cells (Figure 2C,D), reinforcing the proof of their non‐participation in the involved canonical inflammasome pathway.

Therefore, we decided to compare the expression levels of three caspase proteins (caspase1/7 and 8) with that of both GSDME and GSDMD by Western blotting and found that the up‐regulation GSDME‐N was correlating with that of c‐Caspase‐3 c‐Caspase‐7 and c‐Caspase‐8 protein in the treated cell lines (Figure 2E,F), Simultaneously, the JNK’s expression was stable during the whole process while the phosphorylated JNK(p‐JNK) was gradually upgraded (Figure 2E,F), indicating the ROS/JNK pathway might be activated.

### 3.4 Pyroptosis mediated by GSDME is activated by the ROS/JNK signalling in breast cancer cells

**FIGURE 3:**
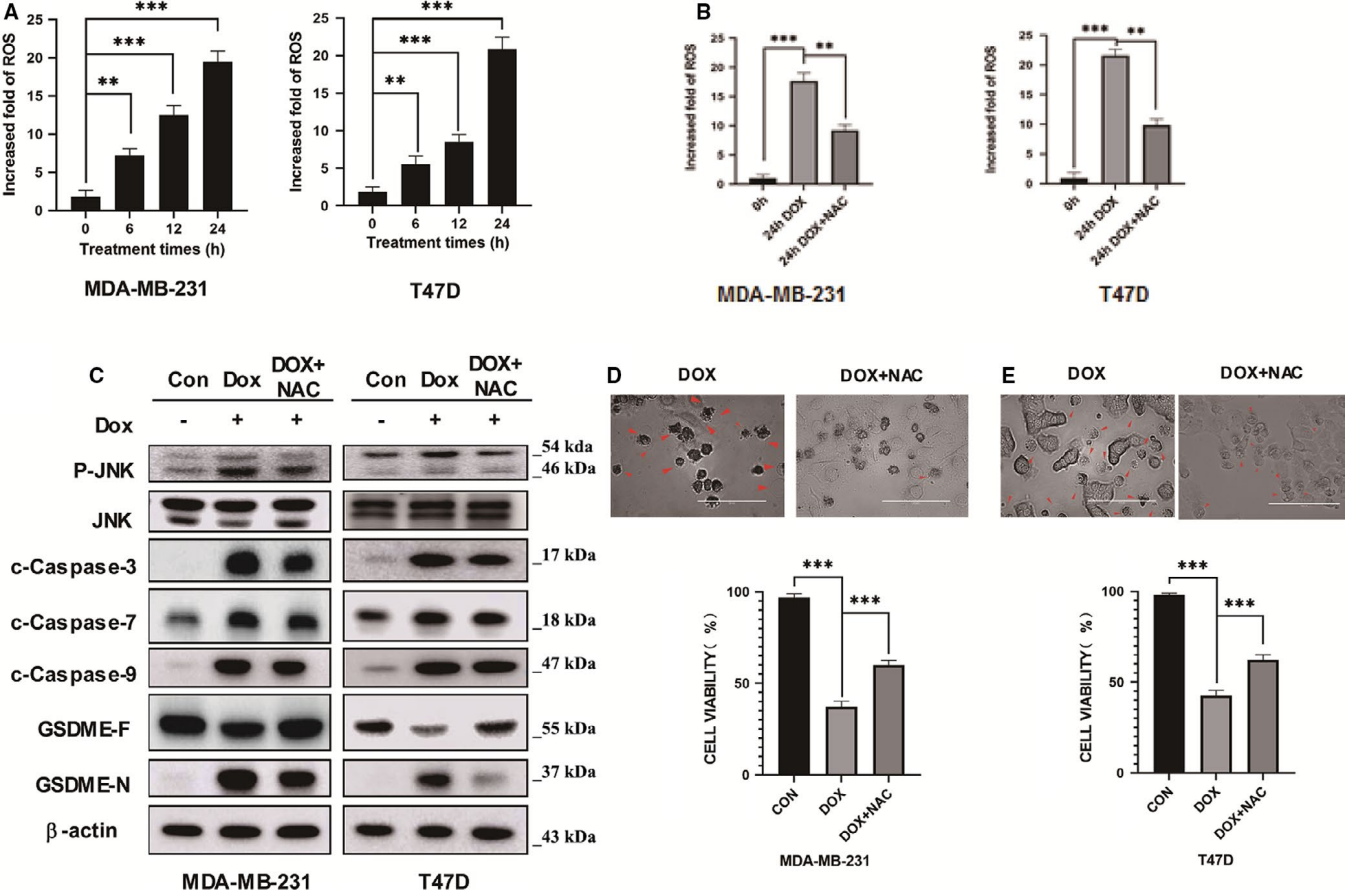
ROS/JNK signalling activates the pyroptosis mediated by GSDME in breast cancer cells. (A) The ROS levels of MDA‐MB‐231 and T47D cells treated with doxorubicin were measured by a ROS assay at different time. (B) The effect of NAC on the ROS levels of MDA‐MB‐231 and T47D cells in the presence of doxorubicin treatment for 12 h was assessed by the ROS assay. (C) The effect of NAC on the protein levels of key factors in pyroptosis in MDA‐MB‐231 and T47D cells with the doxorubicin treatment for 12 h was assessed by the Western blot. (D) and (E) The effect of NAC on the cell viability of MDA‐MB‐231 and T47D cells with the doxorubicin treatment was detected by the CCK8 assay. The representative microscopic images of MDA‐MB‐231 and T47D showed the pyroptosis morphology. Red arrowheads point to the characteristic balloon in the cell membrane. Scale bar, 25 µm. To compare the differences among groups, statistical analyses were monitored using Student’s t‐test or one‐way ANOVA in SPSS 18.0 software (SPSS Inc., Chicago, IL, USA). With: ** = p < 0.01, *** = p < 0.001, ns = p > = 0.05

Pyroptosis reactions were enhanced by caspase‐3 during the mitochondrial apoptosis mainly mediated by ROS/JNK/Bax signalling pathway.^6^ Thus, we monitored the dynamics of the intracellular ROS production in breast cells under DOX treatment. As expected, the ROS level was significantly increased with the DOX treatment after 24 hours (Figure 3A). Then, to evaluate the ROX effect, we reduced the ROS level by using the NAC inhibitor of ROS, and the NAC indeed reduced about 50% intracellular production of ROS in cells under DOX treatment (Figure 3B). We further, analyzed the protein expression level of the key molecules including GSDME‐N, GSDME‐F, JNK, p‐JNK, caspase 3,7 and 9 through a Western blot assay. Our result displayed a collective reduction of the protein expressions of all these key molecules, except for that of the GSDME‐F which was upgraded (Figure 3C). This proves that DOX‐induced phosphorylation of JNK is decreased by NAC application, and linked the DOX‐induced ROS to the JNK pathway. Also, the addition of NAC strongly improved the cell viability in treated cells (Figure 3D,E). Taken together these data evinced that DOX‐induced ROS played an important role in this GSDME‐dependent pyroptosis pathway as well, and c‐Caspase‐3 and c‐Caspase‐9.

### 3.5 DOX‐induced pyroptosis could be rescued

**FIGURE 4:**
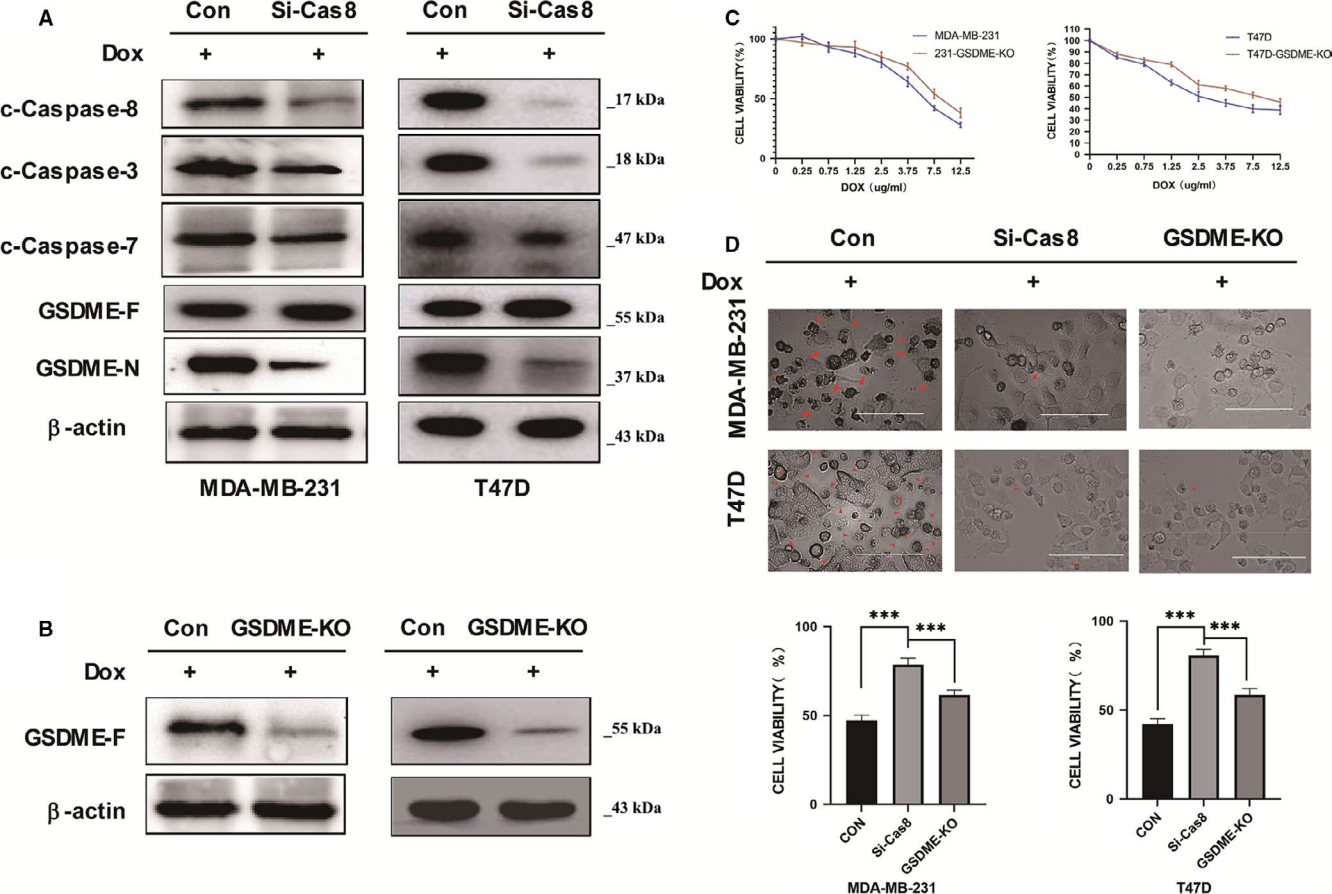
Reduction of GSDME may rescue the DOX‐induced pyroptosis. (A) Knockdown of the Caspase‐8 may decrease the protein levels of key factors including the GSDME‐N. (B) Verification of the GSDME knockout cell lines. (C) Knockout of the GSDME may significantly increase the cell viability of MDA‐MB‐231 and T47D cells under the doxorubicin treatment. (D) Both knockdown of the Caspase‐8 and knockout of the GSDME may rescue the doxorubicin treated cells from pyroptosis. To compare the differences among groups, statistical analyses were monitored using Student’s t‐test or one‐way ANOVA in SPSS 18.0 software (SPSS Inc., Chicago, IL, USA). With: *** = p < 0.001, ns = p > = 0.05

Since the activity of Caspase‐3 is regulated by Caspase‐8, we supposed that modulating the expression of Caspase‐8 might affect cell‐induced pyroptosis reactions.^16^, ^17^ To verify this hypothesis, we knocked down the protein expression of Caspase‐8 with a target‐specific siRNA (si‐Cas8) in two cell lines and detected the protein level of GSDME‐N, GSDME‐F, caspase 3/7/8 under the DOX treatment via Western blot analysis. Interestingly, the knockdown of Caspase 8 resulted in a heavy reduction of Caspase‐3 protein's expression. Likewise, the protein level of Caspase‐7 was lightly dismissed while that of the cleaved GSDME‐N also decreased remarkably when compared to the control cells (Figure 4A). These results showed Caspase‐8 as upstream of Caspase‐3, and Caspase‐7 reaction could play key roles in pyroptosis’ modulation in cancerous cells. To confirm the role of GSDME/DOX‐induced pyroptosis, we knock out the expression of GSDME in MDA‐MB‐231 and T47D cell lines by using the CRISPR/Cas9 technology and evaluated both its DOX‐induced protein expression and cell viability through the Western blot and CCK8 assay, respectively. We found that the protein expression of GSDME‐F was almost inexistent after the CRISPR/Cas9 assay, proof of its successful application and that the cell viability was improved in treated cells with a knocked out GSDME (GSDME‐KO) (Figure 4B,C). Then, using the CCK8 and microscopy assays, we compared the DOX‐induced pyroptosis between si‐Cas8 cell, GSDME‐KO and the control. As displayed by Figure 4D DOX‐induced cell viabilities were ameliorated in both si‐Cas8 and GSDME‐KO cells, as well as the pyroptosis effects, were remarkably mitigated, with most significances in GSDME‐KO cells. Taken together, these results showed that pyroptosis mediated by GSDME in breast cancer cells could be rescued by reducing the protein level of active Caspase‐3, Caspase‐8 or direct inhibition of GSDME.

## 4 DISCUSSION

Unlike necrotic cell death, pyroptosis occurs in a specific and distinguished pathway that is usually connected with innate immunity.^17^, ^18^ Nowadays, researchers have identified three main pathways that mediate the pyroptosis including the Caspase‐1 dependent canonical inflammasome pathway, the Caspase‐4/5/11 dependent non‐canonical inflammasome pathway, and Caspase‐3 dependent pyroptotic pathway. Recently, a study revealed a tight linkage between the cleavage of GSDME and secondary necrosis after apoptosis or pyroptosis.^19^

Doxorubicin is widely recognized as a valuable chemotherapy drug in the nursing of various cancers, however also call ‘red devil’ the drug has many side effects on patients including mouth sores, hair loss, nausea and in certain cases harsh cardiotoxicity which is mainly characterized by pyroptotic reactions. Studies on lung cancer and melanoma demonstrated that DOX treatment strongly induced pyroptosis as side effects.^20^, ^21^ Meanwhile, another study has reported that DOX generated the overflow of mitochondrial ROS, stimulated cellular ROS outflow reactions and caused significant damages in human umbilical vein endothelial cells.^22^ Hyper‐accumulation of ROS also is acknowledged as a serious repressive reaction in various cell signalling pathways, it generates cell death by stimulating the MAPK signalling network.^23^, ^24^ A recent study demonstrated that demethylation of GSDME using decitabine treatment (DAC), induced pyrolysis in mouse colon cancer cells and breast carcinoma cells under chemotherapy nanomedicines.^25^ In breast cancer, the cell survival rate is strongly linked to the expression of the Gasdermin B gene (GSDMB). Reports on HER2‐positive breast cancer indicated that overexpression of GSDMB reduced cell viability and promoted the rate of metastasis. This discovery suggested GSDMB as a novel for the evaluation and prognosis of breast cancer while another study, the known out of GSDME strongly inhibited PTX‐induced pyroptosis in MCF‐7 breast cancer cell.^26^, ^27^

Adriamycin (Doxorubicin) is frequently described as a prior treatment to both early‐stage and metastatic breast cancer; it is generally prescribed with other supplemental drugs.^28^ However, whether the DOX‐induced pyroptosis in breast cancer cells is still puzzling. In this study, we attempted to show demonstrated that DOX chemotherapy can induce severe pyroptosis in human breast cancer cells. Thus, we evaluated the drug‐induced pyroptosis of five clinical chemotherapy drugs in two cultivate breast cancer cell lines namely MDA‐MB‐231 and T47D and we found that DOX treatment generated the strongest pyroptosis effect among all tested drugs (Figure 1A,B). This implies that breast cancer chemotherapy via DOX requires deeper investigation to achieve its greater amelioration.

It has been reported that chemotherapy drugs may induce pyroptosis through Caspase‐3‐dependent cleavage of GSDME^6^. From this perspective, we further analysed the protein expressions of the cleaved GSDME in breast cancer cells under the above five drugs. The highest GSDME cleavage observed in DOX treated cells asserted that DOX induces pyroptosis in breast cancer cells via a Caspase‐GSDME pathway (Figure 1C,D). However, most studies have disclosed the caspase‐1 as the prominent regulator in various cancers, characterized by the discharge of several pro‐inflammatory elements like released pro‐inflammatory actors like NLRP3 and L‐1β into the cytoplasm.^29^ In this inquiry, the protein expression of c‐Caspase‐1 in breast cancer cells under DOX treatment was lightly reduced within 24 hours, while the expression patterns of GSDMD‐F, GSDMD‐C IL‐1β and NLRP3 hardly changed (Figure 2A, B, C and D). This upshot excluded the Caspase‐1‐dependent canonical inflammasome pathway role in breast cancer cell DOX‐induced pyroptosis. Hence, the comparative analysis of the protein expressions of three different caspases indicated that adjoining the expected caspase‐3, the c‐Caspase‐7 and c‐Caspase‐8 protein were also amplified (Figure 2E,F), confirming the enrolment of the caspase‐GSDME pathway in DOX‐induced pyroptosis.

The caspase‐GSDME pathway is usually associated with intracellular ROS production in cancerous cells. Many researchers have targeted the relation between the caspase‐GSDME pathway and ROS signalling, revealing various enlighten results. For example, in melanoma cells, iron‐activated ROS engenders pyroptosis through the Tom20‐Bax‐caspase‐GSDME pathway. The author demonstrated that the caspase‐3 was activated by cytochrome c freed during the oxidation of protein Tom20.^24^ Here, the increased accumulation of ROS in a time‐dependent manner and its reduction were observed during induced ROS inhibition MDA‐MB‐231 and T47D cell lines under DOX treatment (Figure 3A,B). Likewise, the cell viability and imagery essays displayed important improvements and notable abolitions of pyroptosis effects in ROX inhibited cancer cells, respectively. However, protein expression of GSDME‐N, GSDME‐F, JNK, p‐JNK, caspase 3, caspase‐7 and caspase‐9 were all decreased, while that of the GSDME‐F was upgraded (Figure 3C,D and E). These results indicated that ROS actively participated in responsive reactions of the breast cancer cell to DOX treatment and Caspase‐3‐mediated GSDME induced pyroptosis, notably through ROS /JNK pathway.

Base on resemblances between protein expressions of c‐caspase‐3 and c‐caspase‐8 in Caspase‐GSDME pathway essays, we performed two independent experiments to evaluate the role of c‐caspase‐8 in DOX‐induced pyroptosis, since previous studies had emphasized the implication on c‐caspase‐3. In this study, the reduction of the protein expression levels of both the Caspase‐3 and the cleaved GSDME‐N in si‐Cas8 cells revealed the crosstalk between c‐caspase‐8 reactions and that of GSDME (Figure 4A). Also, DOX‐induced cell viabilities were improved in both si‐Cas8 and GSDME‐KO cells as well as the reduction pyroptosis effects, with most significances in GSDME‐KO cells. This encouraging discovery may provide a new strategy to develop clinical therapy for breast cancer (Figure 4B, C, D and E). These results showed that to alleviate the side effects of pyroptosis mediated by GSDME in breast cancer cells researchers can target the protein Caspase‐3, or Caspase‐8, or direct knockdown of GSDME.

**FIGURE 5:**
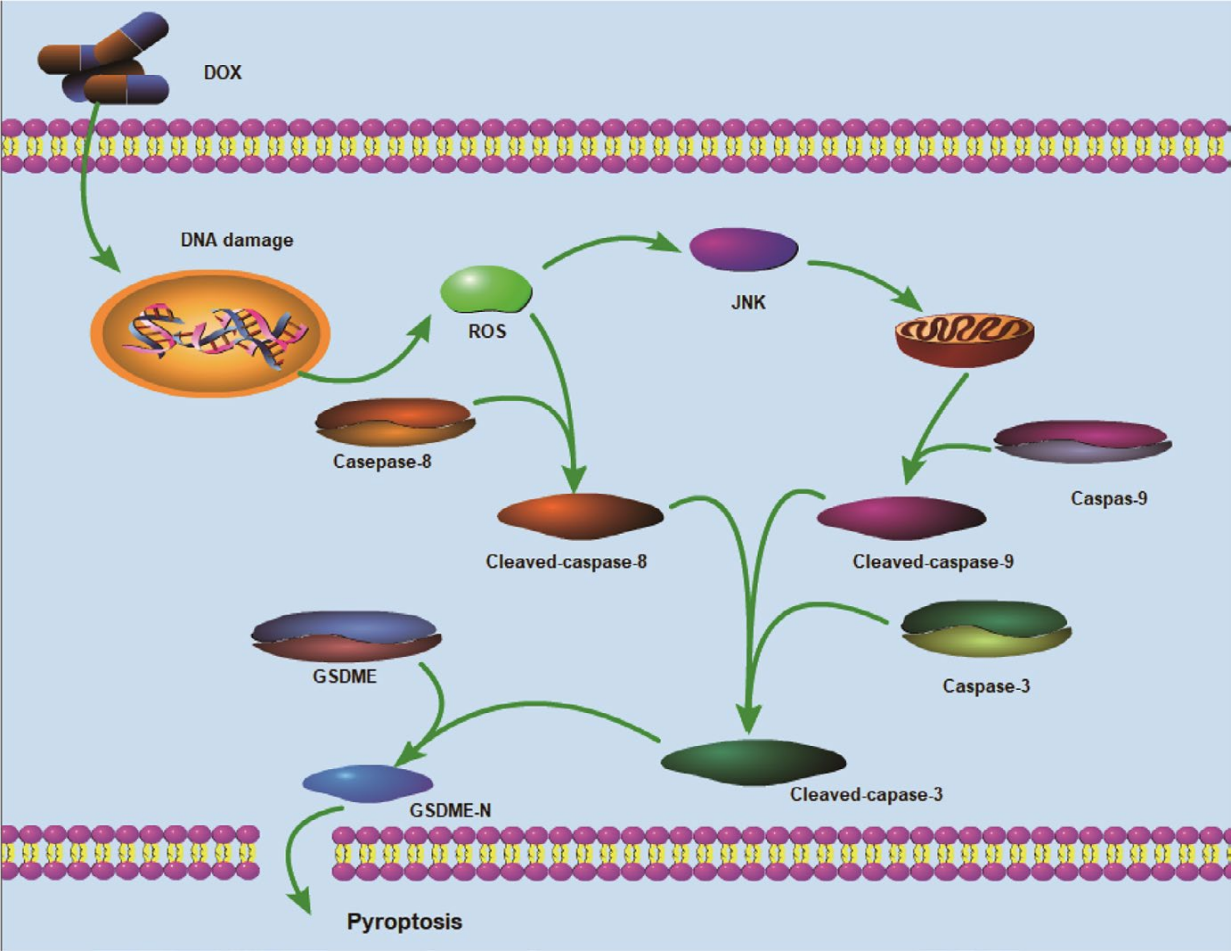
Potential mechanism of DOX‐induced pyroptosis in breast cancer cell

Finally, we can disclose two faces of the ROS /JNK pathway, verified by multiple experimental results. Perhaps, in the two breast cancer cell lines, DOX treatment‐induced ROS accumulation, which subsequently promoted the phosphorylation of JNK into p‐JNK; the latter will further activate the key regulator Caspase‐3 through cascade reaction. On the other hand, DOX‐activated ROS affects the cleavage of Caspase‐8; the c‐Caspase‐8 would promote the cleavage of Caspase‐3, the c‐Caspase‐3 will induce the cleavage of GSDME, and triggered pyroptosis of breast cancer cells (Figure 5).

## 5 CONCLUSIONS

In sum, the present study provided supporting data on DOX‐induced pyroptosis in human cancers, highlighting its effect in human breast cancers for the first time, and confirmed three different targets for DOX‐induced pyroptosis chemotherapy. Therefore, pyroptosis side effects are emerging as ubiquitous in many human cancers; thus, deeper studies are required with a focus on finding novel chemotherapies for pyroptosis in anti‐breast cancer and tumour diseases in general.

## CONFLICT OF INTEREST

The authors declare that there are no conflicts of interest.

## AUTHOR CONTRIBUTIONS

**Ziwen Zhang:** Investigation (lead); Methodology (equal). **Han Zhang:** Investigation (equal); Methodology (equal). **Dongbo Li:** Data curation (equal); Formal analysis (equal); Methodology (supporting). **Xiaoping Zhou:** Software (lead); Validation (lead); Visualization (lead). **Qi Qin:** Resources (lead); Writing‐original draft (lead). **Qingyuan Zhang:** Conceptualization (lead); Funding acquisition (lead); Project administration (lead); Writing‐review & editing (supporting).

## Supporting information

Fig S1

## Data Availability

The data that support the findings of this study are available from the corresponding author upon reasonable request.

## References

[1] McGuire A, Brown J, Malone C, et al. Effects of age on the detection and management of breast cancer. Cancers. 2015;7(2):908‐929. 26010605 10.3390/cancers7020815 PMC4491690

[2] Balasubramanian R, Rolph R, Morgan C, et al. Genetics of breast cancer: management strategies and risk‐reducing surgery. Br J Hosp Med. 2019;80(12):720‐725. 10.12968/hmed.2019.80.12.720 31822191

[3] Anderson BO, Braun S, Lim S, et al. Early detection of breast cancer in countries with limited resources. Breast J. 2003;9:S51‐S59. 12713497 10.1046/j.1524-4741.9.s2.4.x

[4] Kroschinsky F, Stölzel F, von Bonin S , et al. New drugs, new toxicities: severe side effects of modern targeted and immunotherapy of cancer and their management. Crit Care. 2017;21(1):1‐11. 28407743 10.1186/s13054-017-1678-1 PMC5391608

[5] Wang Y, Gao W, Shi X, et al. Chemotherapy drugs induce pyroptosis through caspase‐3 cleavage of a gasdermin. Nature. 2017;547(7661):99‐103. 28459430 10.1038/nature22393

[6] Yu J, Li S, Qi J, et al. Cleavage of GSDME by caspase‐3 determines lobaplatin‐induced pyroptosis in colon cancer cells. Cell Death Dis. 2019;10(3):1‐20. 10.1038/s41419-019-1441-4 PMC6389936 30804337

[7] Aniogo EC, George BPA, Abrahamse H. Phthalocyanine induced phototherapy coupled with Doxorubicin; a promising novel treatment for breast cancer. Expert Rev Anticancer Ther. 2017;17(8):693‐702. 28657372 10.1080/14737140.2017.1347505

[8] Shen X, Wang H, Weng C, et al. Caspase 3/GSDME‐dependent pyroptosis contributes to chemotherapy drug‐induced nephrotoxicity. Cell Death Dis. 2021;12(2):1‐16. 33589596 10.1038/s41419-021-03458-5 PMC7884686

[9] Mai FY, He P, Ye J‐Z, et al. Caspase‐3‐mediated GSDME activation contributes to cisplatin‐ and doxorubicin‐induced secondary necrosis in mouse macrophages. Cell Prolif. 2019;52(5):e12663. 31347748 10.1111/cpr.12663 PMC6797504

[10] Gao J, Qiu X, Xi G, et al. Downregulation of GSDMD attenuates tumor proliferation via the intrinsic mitochondrial apoptotic pathway and inhibition of EGFR/Akt signaling and predicts a good prognosis in non‑small cell lung cancer. Oncol Rep. 2018;40(4):1971‐1984. 30106450 10.3892/or.2018.6634 PMC6111570

[11] Shi J, Zhao Y, Wang K, et al. Cleavage of GSDMD by inflammatory caspases determines pyroptotic cell death. Nature. 2015;526(7575):660‐665. 26375003 10.1038/nature15514

[12] Ding J, Wang K, Liu W, et al. Pore‐forming activity and structural autoinhibition of the gasdermin family. Nature. 2016;535(7610):111‐116. 27281216 10.1038/nature18590

[13] Lu H, Zhang S, Wu J, et al. Molecular targeted therapies elicit concurrent apoptotic and GSDME‐dependent pyroptotic tumor cell death. Clin Cancer Res. 2018;24(23):6066‐6077. 30061362 10.1158/1078-0432.CCR-18-1478

[14] Jorgensen I, Miao EA. Pyroptotic cell death defends against intracellular pathogens. Immunol Rev. 2015;265(1):130‐142. 25879289 10.1111/imr.12287 PMC4400865

[15] Shi J, Gao W, Shao F. Pyroptosis: gasdermin‐mediated programmed necrotic cell death. Trends Biochem Sci. 2017;42(4):245‐254. 27932073 10.1016/j.tibs.2016.10.004

[16] Kamradt MC, Chen F, Cryns VL. The small heat shock protein αB‐crystalline negatively regulates cytochrome c‐and caspase‐8‐dependent activation of caspase‐3 by inhibiting its autoproteolytic maturation. J Biol Chem. 2001;276(19):16059‐16063. 11274139 10.1074/jbc.C100107200

[17] Kroemer G, Galluzzi L, Vandenabeele P, et al. Classification of cell death: recommendations of the Nomenclature Committee on. Cell Death. 2009;16(1):3‐11. 10.1038/cdd.2008.150 PMC2744427 18846107

[18] Galluzzi L, Vitale I, Kroemer G, et al. Molecular mechanisms of cell death: recommendations of the Nomenclature Committee on. Cell Death. 2018;25(3):486‐541. 10.1038/s41418-017-0012-4 PMC5864239 29362479

[19] Rogers C, Fernandes‐Alnemri T, Mayes L, et al. Cleavage of DFNA5 by caspase‐3 during apoptosis mediates progression to secondary necrotic/pyroptotic cell death. Nat Commun. 2017;8(1):1‐14. 28045099 10.1038/ncomms14128 PMC5216131

[20] Tsang W, Chau SPY, Kong SK, et al. Reactive oxygen species mediate doxorubicin induced p53‐independent apoptosis. Life Sci. 2003;73(16):2047‐2058. 12899928 10.1016/s0024-3205(03)00566-6

[21] Yu P, Wang H‐Y, Tian M, et al. Eukaryotic elongation factor‐2 kinase regulates the cross‐talk between autophagy and pyroptosis in doxorubicin‐treated human melanoma cells in vitro. Acta Pharmacol Sin. 2019;40(9):1237‐1244. 30914761 10.1038/s41401-019-0222-z PMC6786479

[22] He H, Wang L, Qiao Y et al. Doxorubicin induces endotheliotoxicity and mitochondrial dysfunction via ROS/eNOS/NO Pathway. Frontiers Pharmacol. 2020;10:1531. 10.3389/fphar.2019.01531 PMC6965327 31998130

[23] Shadel GS, Horvath TLJC. Mitochondrial ROS signaling in organismal homeostasis. Cell. 2015;163(3):560‐569. 26496603 10.1016/j.cell.2015.10.001 PMC4634671

[24] Zhou B, Zhang J‐Y, Liu X‐S, et al. Tom20 senses iron‐activated ROS signaling to promote melanoma cell pyroptosis. Cell Res. 2018;28(12):1171‐1185. 30287942 10.1038/s41422-018-0090-y PMC6274649

[25] Fan J‐X, Deng R‐H, Wang H, et al. Epigenetics‐based tumor cells pyroptosis for enhancing the immunological effect of chemotherapeutic nanocarriers. Nano Lett. 2019;19(11):8049‐8058. 31558023 10.1021/acs.nanolett.9b03245

[26] Hergueta‐Redondo M, Sarrió D, Molina‐Crespo Á, et al. Gasdermin‐B promotes invasion and metastasis in breast cancer cells. PLoS One. 2014;9(3):e90099. 24675552 10.1371/journal.pone.0090099 PMC3967990

[27] Shi YJCJCB. GSDME influences sensitivity of breast cancer MCF‐7 cells to paclitaxel by regulating cell pyroptosis. Chin J Cancer Biother. 2019;26(2):146‐151. 10.3872/j.issn.1007-385X.2019.02.002

[28] Lao J, Madani J, Puértolas T, et al. Liposomal doxorubicin in the treatment of breast cancer patients: a review. Journal of Drug Delivery. 2013;2013:1‐12. 10.1155/2013/456409 PMC3619536 23634302

[29] Xia X, Wang X, Cheng Z, et al. The role of pyroptosis in cancer: pro‐cancer or pro‐“host”? Cell Death Dis. 2019;10(9):1‐13. 10.1038/s41419-019-1883-8 PMC6733901 31501419

