# Supplementary figures and images for: Caspase‐3‐mediated GSDME induced Pyroptosis in breast cancer cells through the ROS/JNK signalling pathway
Source: J Cell Mol Med. 2021 Aug 8;25(17):8159–68. doi: 10.1111/jcmm.16574 (PMC8419174; doi:10.1111/jcmm.16574)

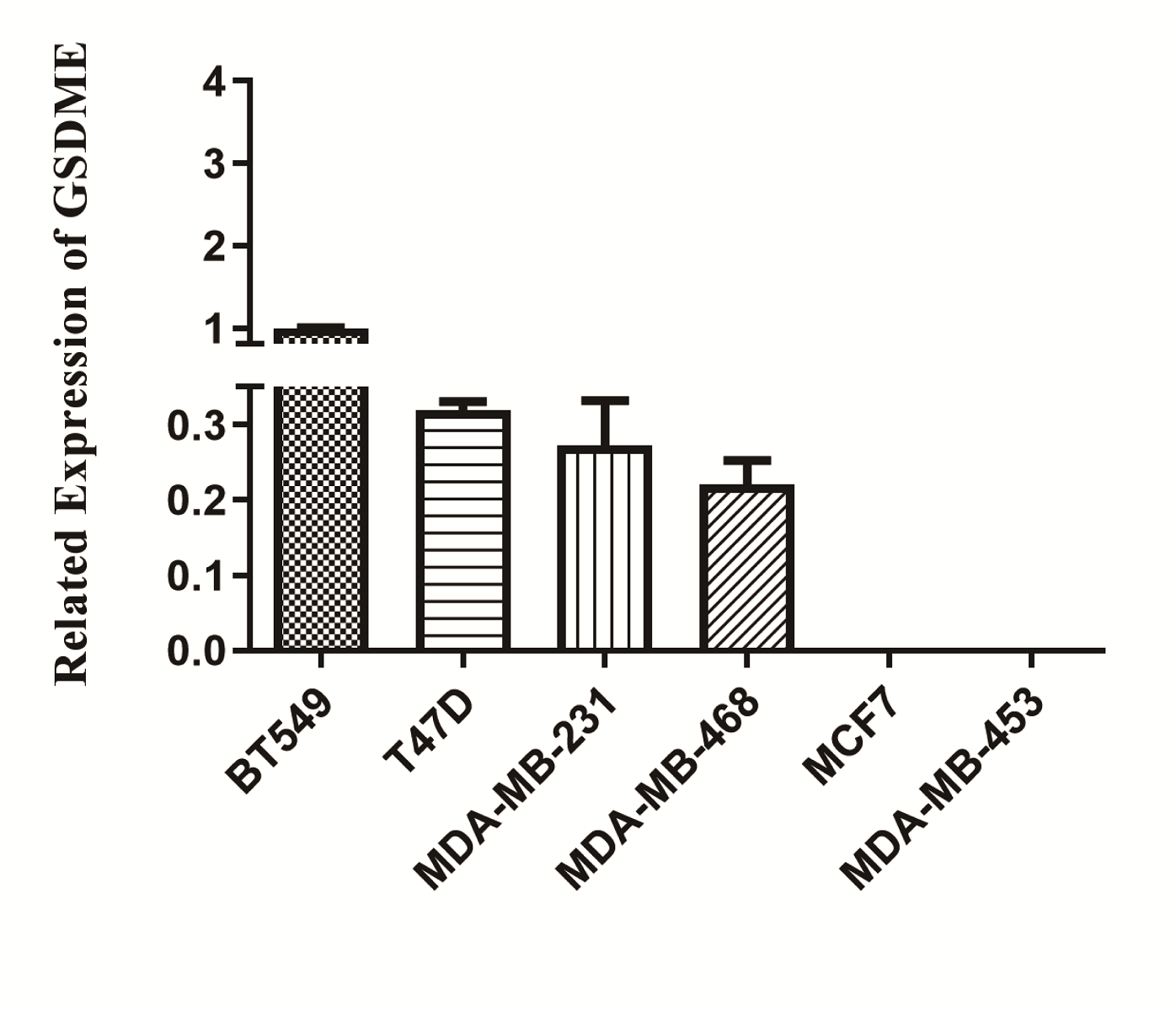

Supplement: Supplementary file 1 — Fig S1 [file JCMM-25-8159-s001.png]
